# Implementing carotid ultrasound and Holter monitoring through telemedicine-based training in a stroke unit in Lusaka, Zambia

**DOI:** 10.1186/s42466-025-00448-2

**Published:** 2025-11-14

**Authors:** Burc Bassa, S. Braun, K. Aydin, A. Rindermann, M. Luchembe, M. Nthere, D. Mwansa, L. Yankae, C. Namangala, M. Bwalya, M. Belau, D. Saylor, U. Meyding-Lamadé

**Affiliations:** 1 Department of Neurology at Krankenhaus Nordwest, Frankfurt, Germany; 2 Department of Neurology, University Teaching Hospital, Lusaka, Zambia; 3 Department of Cardiology at Krankenhaus Nordwest, Frankfurt, Germany; 4 Institute of Medical Biometry and Epidemiology, University Medical Center Hamburg-Eppendorf, Hamburg, Germany; 5 Department of Neurology, University of North Carolina, Chapel Hill, NC USA; 6 Department of Neurology, University of Heidelberg, Heidelberg, Germany

## Abstract

**Background and aims:**

Stroke remains a leading cause of mortality and disability in many low- and middle-income countries, where access to diagnostic and treatment resources is often severely constrained. This pilot study investigated the feasibility of telemedicine-based training to integrate carotid artery ultrasound and Holter monitoring into routine diagnostic practices at the stroke unit of the University Teaching Hospital (UTH) in Lusaka, Zambia.

**Methods:**

Five neurology residents at the University Teaching Hospital in Zambia, without prior clinical experience in carotid artery ultrasound, received remote online training sessions. Subsequently, they were divided into two groups: the first conducted practical examination sessions under on-site supervision of a stroke neurologist, while the second was remotely supervised via screen sharing by a stroke neurologist from Krankenhaus Nordwest in Frankfurt (KHNW), Germany. Handheld portable ultrasound probes (Butterfly IQ+) were used for the examinations. Following the training, each group of residents performed 50 extracranial ultrasound examinations in acute ischemic stroke patients at the UTH stroke unit. Each examined patient was re-examined in a separate session by an experienced stroke neurologist, who was blinded to the results of the residents’ examination. The agreement between raters in the assessment of carotid stenosis was assessed using Cohen’s kappa (κ), a statistical measure that evaluates interrater reliability for categorical items. Similarly, 26 stroke nurses at UTH were trained in Holter monitoring exclusively through video tutorials, without hands-on practice. They recorded 30 Holter examinations on subsequent acute ischemic stroke patients. The quality of the recordings was subsequently compared to 30 Holter recordings from consecutive patients at the stroke unit of KHNW. A cardiologist, blinded to the origin of the recordings, evaluated their quality on a scale of 1 to 10, and the results were analyzed using Welch’s t-test. All participants completed multiple-choice assessments to evaluate their theoretical knowledge, along with a feedback survey on the training program.

**Results:**

50 patients underwent bilateral carotid artery ultrasound examination, split evenly between the direct and remotely supervised groups. Both groups achieved a high rate of concordance with an experienced stroke neurologist. The directly supervised group achieved 86% concordance for ICA stenosis and 88% for plaque detection, while the remotely supervised group achieved 80% and 84%, respectively. Holter recordings from UTH demonstrated higher quality than those from the stroke unit at KHNW (*p* < 0.01). Most participants reported enhanced confidence and knowledge, though over 60% preferred face-to-face training formats.

**Conclusion:**

Innovative telemedical training approaches offer a transformative solution for addressing diagnostic and infrastructure challenges in stroke care, particularly in resource-constrained healthcare settings. Comprehensive research is crucial to optimize these programs and enhance medical capabilities across diverse healthcare environments.

## Background and aims

Stroke is one of the leading causes of mortality and long-term disability worldwide, with a disproportionately higher burden in low- and middle-income countries (LMICs). These regions, particularly in Sub-Saharan Africa, South Asia, and Latin America, experience a rising incidence of stroke due to demographic shifts, increasing prevalence of cardiovascular risk factors, and limited preventive measures [1, 2].

In LMICs, the healthcare infrastructure frequently lacks essential diagnostic tools critical for effective stroke management. Advanced diagnostic technologies, such as computed tomography (CT) and magnetic resonance imaging (MRI) are scarce or entirely unavailable outside major urban centers. Similarly, specialized stroke workup diagnostics—such as carotid artery ultrasound and Holter monitoring, which are essential for detecting high-risk conditions including carotid artery stenosis and atrial fibrillation—remain unavailable in many settings.

Therapeutic resources in LMICs also face severe constraints. The acute management of stroke, notably thrombolytic therapy, requires rapid administration and precise diagnosis, but these conditions are seldom achievable in resource-limited environments due to shortages in trained personnel, medication availability, and infrastructure deficits. Additionally, the absence of dedicated stroke units and trained stroke care teams further worsens patient outcomes, increasing mortality and disability rates [3, 4].

Moreover, the chronic phase of stroke management, encompassing rehabilitation and secondary prevention strategies, remains significantly underdeveloped. Rehabilitation services, including physiotherapy, occupational therapy, and speech therapy, are minimally available or entirely absent in many LMIC settings, greatly affecting patient recovery and quality of life [5–7].

Addressing these challenges necessitates innovative, cost-effective, and sustainable solutions such as telemedicine-based training and task-shifting strategies to empower healthcare professionals and caregivers. Enhancing diagnostic capabilities through remote educational approaches and improving the availability of affordable medical technologies can substantially mitigate current disparities. Comprehensive investment in healthcare infrastructure and personnel training is critical to reducing the burden of stroke in LMICs.

Stroke is a growing health concern in Sub-Saharan Africa (SSA), driven by urbanization, aging populations, and increasing prevalence of non-communicable diseases. Unlike in high-income regions, stroke in SSA often affects younger adults, with ischemic strokes being the most common subtype. Hypertension is the leading modifiable risk factor, followed by diabetes, dyslipidemia, and lifestyle factors such as smoking and inactivity. Epidemiological studies from Sub-Saharan Africa and other low-resource settings highlight the growing impact of stroke among younger adults, often with limited diagnostic access and differing etiological profiles [3, 8].

Internal carotid artery (ICA) atherosclerosis and cardioembolic stroke are two of the leading causes of stroke in Western countries, but their prevalence is less understood in LMICs. Atrial fibrillation (AF) is the most frequent arrhythmia reported in AIS patients and a common cause of cardioembolic stroke. Yet, 25–62% of people with AF have only transient arrhythmias, such as paroxysmal AF, necessitating prolonged cardiac rhythm monitoring to increase detection probability [9]. Ultrasound of the carotid artery and Holter monitoring are two essential diagnostic procedures to determine the stroke etiology and guide optimal secondary prophylaxis. Many people with AF and stroke benefit from anticoagulation, whereas, in people with stroke and high-grade stenosis of the ICA, endarterectomy may be warranted [10, 11].

Zambia is a large, sparsely populated country of more than 20 million people in southern Africa and is classified as a lower middle-income country by the World Bank. In 2019, the age-standardized mortality rate from stroke was 162.1 per 100,000 population, second only to HIV/AIDS as the leading cause of death. Previous studies have suggested that the proportion of hemorrhagic stroke may be higher than in many Western countries. However, a substantial percentage of patients did not undergo neuroimaging, which limits the ability to draw definitive conclusions. The University Teaching Hospital (UTH), located in Lusaka, is a tertiary public healthcare center and a national referral hospital with over 1,700 beds. The country’s first standardized stroke unit was launched at UTH in October 2023 [12, 13].

This study assessed the feasibility of a predominantly telemedicine-based training approach to integrate carotid artery ultrasound and Holter monitoring into routine clinical practice at the stroke unit of the University Teaching Hospital in Zambia. We additionally evaluated the residents’ experiences throughout the course and the challenges inherent to delivering telemedical training in resource-limited settings.

## Methods

### Study design, setting, and participants

This prospective, pilot study was conducted at the University Teaching Hospital (UTH) in Lusaka, Zambia, and at Krankenhaus Nordwest (KHNW) in Frankfurt, Germany. The protocol was approved by the University of Zambia Biomedical Research Ethics Committee (IRB00001131) and the State Medical Association of Hesse (2023-3350-evBO). Patients were recruited from the UTH Stroke Unit; eligible participants were adults admitted with acute ischemic stroke confirmed by CT, while those with hemorrhagic stroke or stroke mimics were excluded.

Five neurology residents at UTH, without any prior experience in carotid artery ultrasound, received telemedical training on theoretical aspects of the examination. The training consisted of three modules, each consisting of 45 min of theoretical training followed by 15 min for answering questions. (Table 1).

Subsequently, they were divided into two groups. In the first group, two residents performed extracranial carotid ultrasound examinations under the direct supervision of a stroke neurologist (SB) on-site. In the second group, three residents conducted the examinations with real-time remote supervision via screen-sharing by a stroke neurologist (BB) from KHNW in Frankfurt, Germany. Both neurologists who provided the training were board-certified specialists in neurology trained in Germany, each with many years of experience in stroke unit care.

The examinations were conducted with portable handheld ultrasound probes (Butterfly IQ+), which connect to a smartphone or tablet.

Because two of the participating residents, one in either group, had basic prior experience with point-of-care cardiac ultrasound, the training sessions were individually tailored to their existing skill levels. Each neurology resident received personalized instruction based on prior familiarity with portable ultrasound, with total training time averaging three to five hours per participant.

**Table 1:** Theoretical content covered in the training modules

Module 1: Introduction to Carotid Ultrasound
Anatomy and Physiology Ultrasound Physics and Principles Blood flow dynamics and hemodynamics in normal carotid arteries Equipment and Settings, Scanning Techniques
Module 2: Carotid Artery Ultrasound Examination
Examples of normal and pathologic examinations Image optimization techniques Diagnostic Criteria, Carotid Ultrasound Protocols Carotid artery stenosis: grading and hemodynamic assessment
Module 3: Reporting, Limitations, Clinical Correlation
Structured reporting Limitations of point-of-care probes and artifact recognition Medical and surgical management options

After the training period, each group independently examined 25 consecutive acute ischemic stroke patients admitted to the UTH. All patients were examined bilaterally, and each ICA was graded into one of five categories according to The Society of Radiologists in Ultrasound consensus criteria [14]. The examination was then repeated by a stroke neurologist (SB), who was blinded to the results of the residents’ examination. The overlap in identifying the same category of carotid artery stenosis was tested using Kappa statistics.

Similarly, 26 stroke-unit nurses at UTH without previous experience in Holter monitoring underwent remote training. A video was prepared showing the recording procedure, including correct lead placement, artifact recognition, and clinical indications, which was subsequently distributed through a messaging and communication app. Subsequently, online sessions were conducted by cardiologists from KHNW to answer questions that arose. No onsite training was provided. After the training period, four Holter monitors (ECG Time Holter Recorder, Medset^®^) were provided and the stroke unit nurses recorded Holter ECGs in 30 subsequent AIS-patients.

These examinations were pseudonymized, encrypted and transferred through a secured VPN connection to a cloud server in Germany, where they were compared to the same number of examinations from AIS-patients from the KHNW stroke unit. A cardiologist reviewed the recordings blinded to their source and rated based on several factors, including the number of leads with artifacts and the duration of artifacts. The quality of the recordings was rated on an ordinal rating scale with 10 being excellent and 1 indicating continuous artifacts in all three recording leads. The results were compared using the Welch’s t test.

To evaluate the teaching approach, residents and stroke nurses were assessed using pre- and post-training questionnaires, each consisting of 10 multiple-choice questions focused on carotid artery ultrasound for doctors and Holter monitoring for nurses, respectively (Supplement 1).

For all training sessions the communication platform Zoom (Zoom Video Communications, Inc and WhatsApp Messaging Service, Inc) was used. Informed consent was obtained from all participating patients at both institutions.

### Statistical analyses

The agreement between each resident’s carotid artery classification and that of the stroke neurologist (SB) was assessed using Cohen’s kappa (κ), a statistical measure that evaluates interrater reliability for categorical items. For the analysis of differences in Holter monitoring signal classification, we utilized Welch’s t-test. To analyze the impact of the training on theoretical knowledge, we employed the Wilcoxon signed-rank test. All analyses were performed using STATA MP, version 18.

## Results

Bilateral extracranial carotid Doppler ultrasounds were performed in a total of 50 patients, with 25 examinations conducted by the directly supervised group and 25 by the remotely supervised group.

Since each patient had both their ICAs evaluated, each patient contributed two examinations, resulting in a total of 100 carotid ultrasound examinations. While assessing ICA stenosis, the directly supervised group achieved concordant examination results with the stroke neurologist (SB) in 43 out of 50 examinations (86%), whereas the remotely supervised group showed concordance in 40 out of 50 cases (80%).

When assessing the presence of plaques in the ICA and CCA, concordance was achieved in 44 of 50 examinations (88%) in the directly supervised group and in 42 examinations (84%) in the remotely supervised group.

The differences occurred exclusively in the categories “normal” and “<50% stenosis”, which are characterized by unchanged blood flow velocities but differ in whether a stenosis is visible in B-mode imaging. Importantly, in both groups no case of high-grade stenosis or carotid artery occlusion was missed. The agreement between assessments was confirmed by the Kappa statistic (Table 2).

**Table 2:** Classification of ICA stenosis the direct and remotely supervised groups

	Direct Supervision		*p*-value	Remote Supervision		*p*-value
	*n*	%		*n*	%	
**Carotid ultrasound**			< 0.001			< 0.001
Same classification	43	86.0		40	80.0	
Different classification	7	14.0		10	20.0	
Presence of Plaques*			< 0.001			< 0.001
Same classification	44	88.0		42	84.0	
Different classification	6	12.0		8	16.0	

*n*, quantity; %, proportion, * The presence of plaques in the CCA bulb and ICA were assessed in a binary manner, classified as either present or absent

Similarly, the recording quality of 30 Holter recordings from UTH was blindly compared to 30 consecutive recordings from the stroke unit of KHNW. The average recording quality at UTH was 9.0 (± 2.3), compared to 6.9 (± 3.2) at KHNW. The median scores were 10 for UTH and 6 for KHNW, indicating a higher overall recording quality at UTH (*p* < 0.05). The results of the Holter recording quality are summarized in Table 3.

**Table 3:** Quality of Holter exams

	KHNW Germany	UTH Zambia	Difference	*p*-value
**Holter recording quality**			-2,1	0.005
Mean	6.9	9.0		
Median	6	10		
Q1, Q3	6, 10	10, 10		
Min, Max	1, 10	1, 10		
*n*	30	30		

*n*, quantity; SD, standard deviation; Q1, first quartile; Q3, third quartile; Min, minimum; Max, maximum

All participants completed a multiple-choice questionnaire both before and after the online training period. Both nurses and neurology residents demonstrated improved theoretical knowledge in their respective training fields. The results of the pre- and post-training theoretical knowledge assessments are summarized in Table 4.

**Table 4:** Pre- and Post-Training assessment of theoretical knowledge

	Nurses		*p*-value	Doctors		*p*-value
	Pre-Training *N* = 26	Post-Training *N* = 24		Pre-Training^a^*N* = 5	Post-Training^b^*N* = 5	
**Holter monitor**			< 0.001			
Mean (SD)	5.8 (1.4)	7.2 (1.4)				
Median	6	7				
Q1, Q3	5, 7	7, 8				
Min, Max	3,8	3,10				
**Carotid ultrasound**						0.375
Mean (SD)				5 (1.9)	6.2 (0.8)	
Median				6	6	
Q1,Q3				3, 6	6,7	
Min, Max				3, 7	5, 7	

*n*, quantity; %, proportion; SD, standard deviation; Q1, first Quintile; Q3, third quintile; Min, minimum; Max, maximum

^a^ ten questions to answer; ^b^ seven questions to answer

**Fig. 1:**
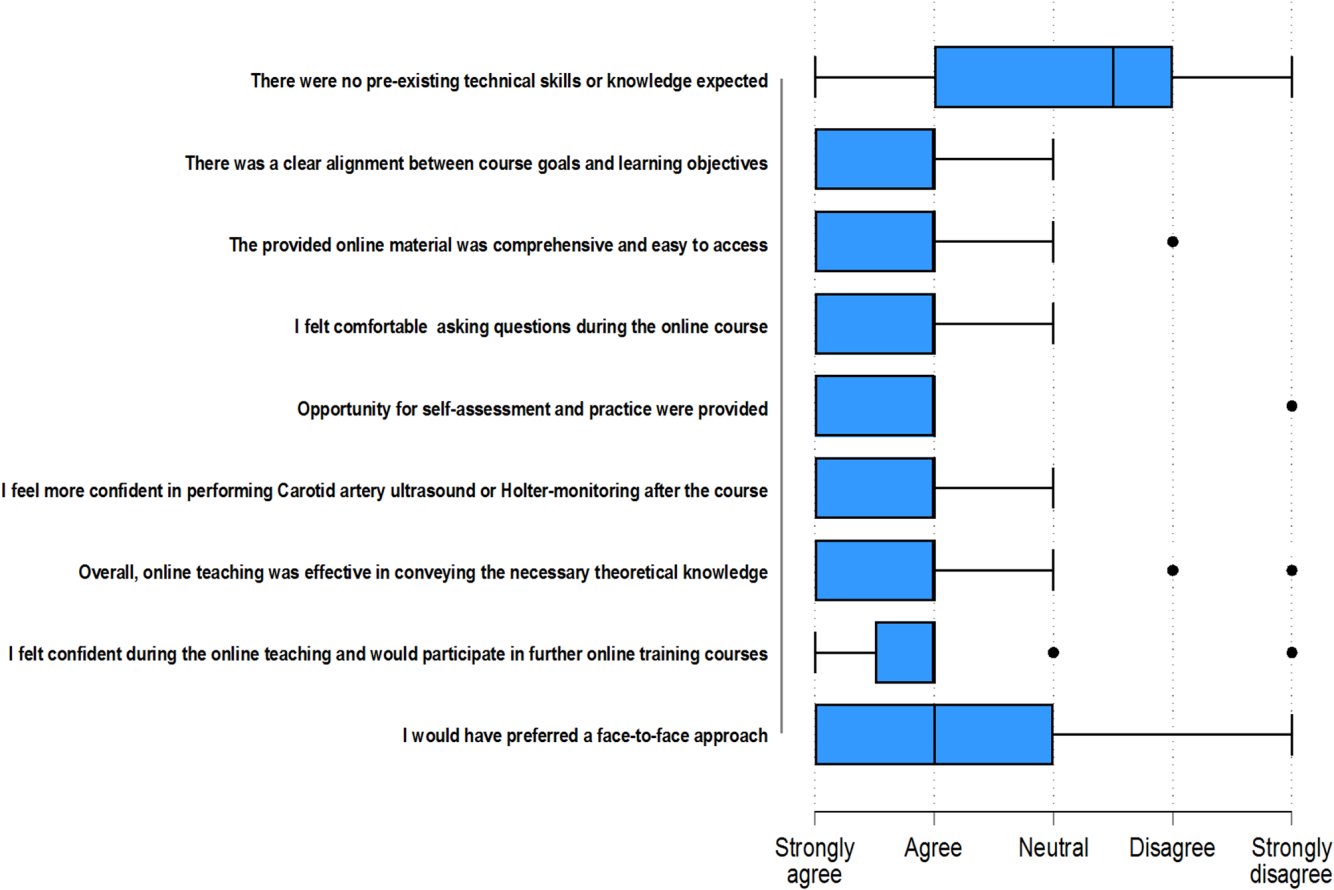
Evaluation of the online training program by participants

At the end of the training period, participants provided feedback on various aspects of the online training. Most participants felt confident in asking questions during the online course. The majority of participating nurses (24 out of 26) reported feeling more confident in performing Holter monitoring after completing the course. While over 80% expressed willingness to participate in additional online training sessions, more than 60% indicated a preference for a face-to-face approach. (Figure 1)

## Discussion

Our pilot study demonstrated the feasibility of a predominantly remote training program for implementing stroke workup diagnostics in resource limited settings.

The limitations in completing a comprehensive stroke workup in LMICs stem from various factors, including shortages in healthcare infrastructure, limited training opportunities, and limited access to essential diagnostic tools [3, 15]. In resource-limited settings, remote training programs offer innovative and practical solutions to address the scarcity of local training opportunities and on-site expertise, effectively bridging critical gaps in capacity building.

In addition to facilitating more widespread access for healthcare providers, remote training programs allow on-site training with locally available resources and represent time- and cost-effective alternatives to training programs abroad. Allowing asynchronous virtual access to some or all training modules facilitates continuous learning and skill refinement through ongoing access to educational materials. Providing expert support beyond the training phase is essential for sustainable local capacity building. Through continuous virtual mentorship and guidance, specialists can engage in discussions with local practitioners about challenging cases. Similar to other implementation studies in stroke-unit settings, our findings underline the feasibility of structured technology-supported training programs to build local capacity [16].

However, the remote training approaches also encounter many practical limitations. Most importantly, many LMICs face challenges with their infrastructure, such as slow and unreliable internet connections or frequent power outages. These factors can significantly impede remote training efforts. In particular, for online supervised live examinations, a reliable and fast internet connection is indispensable [17].

Moreover, in our study, over half of the participants indicated a preference for face-to-face training. The reasons underlying this preference warrant further investigation to identify potential barriers or areas for improvement in remote training formats.

## Conclusions

Remote training programs offer a promising approach to address infrastructure and training gaps in resource-limited settings. They may be a cost-effective and scalable means of delivering advanced training and integrate these diagnostic tools into routine stroke care, particularly in resource-limited settings. However, further research is needed to identify the key factors contributing to their success and to address potential limitations. Such studies could inform the development of more effective and tailored remote training initiatives, ultimately improving patient care across diverse healthcare settings.

## Data Availability

Data are available upon reasonable request.
